# Recent Advances of Plasmonic Nanoparticles and their Applications

**DOI:** 10.3390/ma11101833

**Published:** 2018-09-26

**Authors:** Jianxun Liu, Huilin He, Dong Xiao, Shengtao Yin, Wei Ji, Shouzhen Jiang, Dan Luo, Bing Wang, Yanjun Liu

**Affiliations:** 1 Department of Electrical and Electronic Engineering, Southern University of Science and Technology, Shenzhen 518055, China; liujianxun@hust.edu.cn (J.L.); hehl@mail.sustc.edu.cn (H.H.); d_xiao@outlook.com (D.X.); 13589831680@163.com (S.Y.); loud@sustc.edu.cn (D.L.); 2 Wuhan National Laboratory for Optoelectronics and School of Physics, Huazhong University of Science and Technology, Wuhan 430074, China; 3 School of Information Science and Engineering, Shandong University, Jinan 250000, China; jiwww@sdu.edu.cn; 4 School of Physics and Electronics, Shandong Normal University, Jinan 250014, China; jiang_sz@126.com

**Keywords:** plasmonic nanoparticles, upconversion luminescence, chiral metasurfaces

## Abstract

In the past half-century, surface plasmon resonance in noble metallic nanoparticles has been an important research subject. Recent advances in the synthesis, assembly, characterization, and theories of traditional and non-traditional metal nanostructures open a new pathway to the kaleidoscopic applications of plasmonics. However, accurate and precise models of plasmon resonance are still challenging, as its characteristics can be affected by multiple factors. We herein summarize the recent advances of plasmonic nanoparticles and their applications, particularly regarding the fundamentals and applications of surface plasmon resonance (SPR) in Au nanoparticles, plasmon-enhanced upconversion luminescence, and plasmonic chiral metasurfaces.

## 1. Introduction

Surface plasmon resonance (SPR) is the interaction between electromagnetic fields and free electrons in metals. The free electrons in metals can be excited by electrical components of light to produce collective oscillations. There are two modes formed by such electron oscillations. One is the surface plasmon–polariton (SPP), which can propagate along the metal/dielectric interfaces. The other one is the localized surface plasmon resonance (LSPR), which is confined in a very small volume around an isolated nanoparticle/nanostructure. In both cases, the incident electromagnetic field can be localized at a deep subwavelength scale with a remarkable enhancement of the local field, leading to a broad range of applications in the fields of biology, chemistry, energy, and information [1,2,3,4,5,6,7]. Typical applications in bioimaging [8], sensing [2,9,10], surface-enhanced Raman scattering (SERS) [11,12,13,14], chemical reactions [15], and metasurface [16] have been reported based on the unique characteristics of plasmonics. The significant influence of plasmonics on nanoscience and nanotechnology lies in three major points. (1) Plasmons can resonate with light at a particular frequency to a great degree of freedom; for example, 0D metallic nanoparticles (NPs) and nanorods (NRs) [2,17], one-dimensional (1D) nanowires [18], and two-dimensional (2D) metasurfaces can be used to highly control/utilize LSPR to achieve the selective absorption or reflection of light. (2) Plasmons can produce an enormous enhancement of the light intensity [11]. (3) Plasmons can trap electromagnetic energy in deep subwavelength volumes/areas/lengths [19,20,21].

Early recognition of LSPR came from the selective absorption of metallic NPs in the visible light range [2]. The theoretical description can be well treated by the free electron gas model. Thus far, theoretical advances of plasmonics have led to the great development of design for plasmonic materials and applications of various plasmonic devices. For example, the plasmon effect based on noble metal NPs is applied to refractive index sensing, SERS, and the enhancement of solar energy conversion [5]. Through artificially designing the plasmon resonance with the specific geometries and frequencies, metal NPs can be also used to modulate the luminescence of the emitting nanomaterials. A typical example is to utilize LSPR to enhance the luminescence of the upconversion materials. With the advanced development of nanotechnology, the production and performance of noble metal nanodevices have been rising to an unprecedented height. In addition, the advances in nanoscience and nanotechnology have provided a new platform to study the interaction of plasmonic nanostructures with circularly polarized light for spin–orbit photonics [2]. In this review, we summarize the recent research progress and achievements on LSPR, particularly including: (1) fundamentals and applications of LSPR in Au NPs, (2) plasmon enhanced upconversion luminescence, and (3) plasmonic chiral metasurfaces.

## 2. Fundamentals and Applications of Plasmonics in Au NPs

### 2.1. Fundamentals

**Figure 1:**
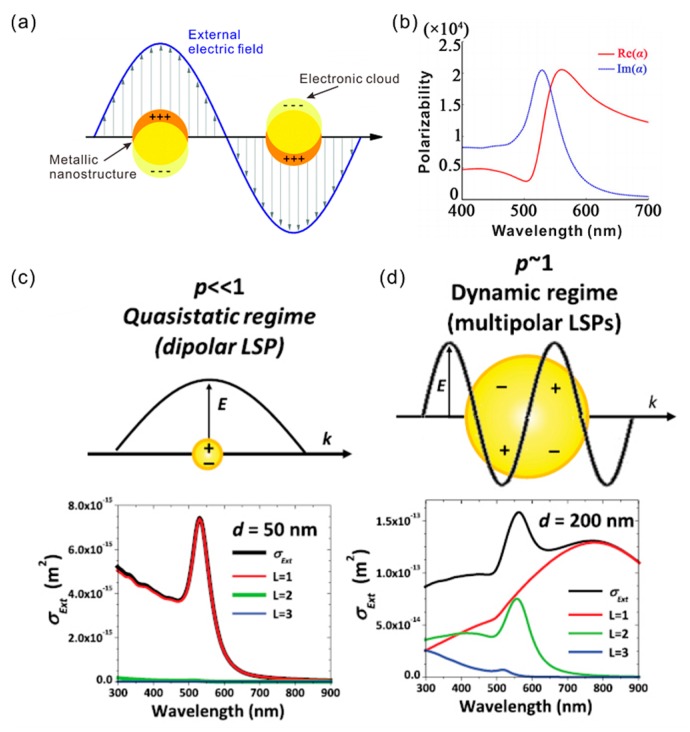
(**a**) Graphical illustration of localized surface plasmon resonance (LSPR) in metallic nanoparticles (NPs). (**b**) The polarizability curve of Au NPs with a diameter of 40 nm in water. (**c**,**d**) Contribution of dipolar (red), quadrupolar (green), and octupolar (blue) LSPR to the *σ_Ext_* (black) in Au NPs with size of 50 nm (**c**) and 200 nm (**d**). Adapted from [2], with permission from © 2017 IOP Publishing.

**Figure 2:**
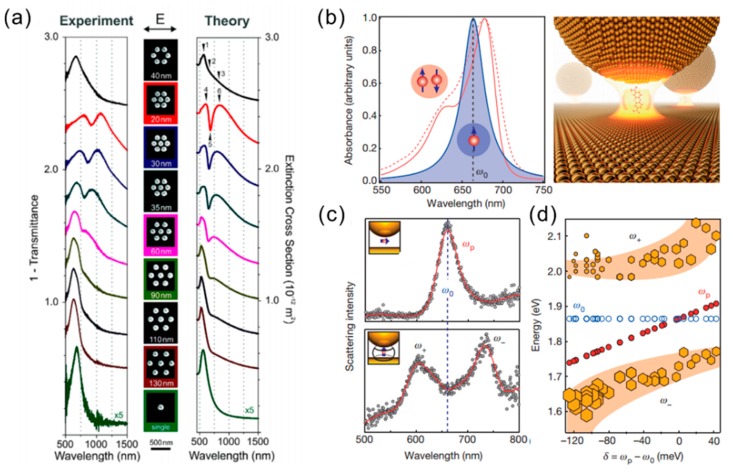
(**a**) Extinction spectra of a gold monomer, a gold hexamer, and gold hepatmers with different interparticle gap separations. Adapted from [24], with permission from © 2010 American Chemical Society. (**b**) Plasmonic nanocavity containing a dye molecule. The schematic diagram illustration of a methylene-blue molecule in cucurbit[7]uril, in the NP on-mirror geometry. (**c**) Scattering spectra resulting from isolated NP on-mirror according to the orientation of the emitter (the methylene-blue dye; see insets). Split peaks result from the strong interaction between the emitter and the plasmon. (**d**) Resonant positions of methylene-blue, plasmon, and hybrid modes as a function of extracted detuning. Adapted from [25], with permission from © 2016 Springer Nature.

In order to understand the physical mechanism of surface plasmons, it is necessary to enlighten the model of the resonant oscillation of the free electrons in metals (Figure 1). Under the excitation of an external electric field, the motion of free electrons in metal NPs can be described by the following equation [1]:

(1) $$m\frac{d^{2}x}{dt^{2}}+m\gamma \frac{dx}{dt}=-eE_{0}\exp\left(-i\varepsilon t\right),$$

where *m*, *γ*, *ω*, and *E*_0_ are the electron mass, damping constant, frequency, and amplitude of the external electric field, respectively. To include the size effect on the optical permittivity of metals, a well-known Drude–Lorentz model has been successfully developed. For the isolated metallic NPs with the size comparable to the incident wavelength, the generated electrons inside the NPs are considered as an ideal gas of non-interacting charged particles under Newton’s second law. The frequency-dependent Drude permittivity of metal NPs can be written as:

(2) $$\varepsilon \left(\omega \right)\;=\varepsilon_{r}+i\varepsilon_{i}=1-\frac{\omega_{p}^{2}}{\omega \left(\omega +i\gamma \right)},$$

where *ω*_p_ is the plasmon frequency, which is close ~10^16^ s^−1^. When γ << *ω*, Equation (2) becomes *ε*(*ω*) *≈* 1 − *ω*_p_^2^/*ω*^2^. Thus, the dielectric constant *ε*(*ω*) can be negative once *ω* < *ω*_p_, and there is a strong interaction between metal and incident electromagnetic waves. In contrast, when *ω* > *ω*_p_, metal is just a conventional dielectric material for an incident electromagnetic field. For the case of metallic NPs with a size far less than the incidence wavelength, the interband transitions will play a major role. Equation (2) is a subset condition of the Drude–Lorentz model, which can be used to explain the physical mechanism of surface plasmons. The Drude–Lorentz model can work well for the case of isolated NPs. There are also other approaches that can provide similar results to Equation (2). For example, the permittivity of isolated metal NP suspended in a medium has been well described by Maxwell–Garnett approach. The permittivity of the connected NPs has also been explained by Bruggeman approach. However, when the NPs are adjacent to each other or in an array arrangement, the electric fields generated from NPs will be coupled each other. Depending on the distance between the NPs, geometry, and light polarization, plasmonic resonances of plasmonic devices (or structures) show the hybrid modes. Typical examples are the Fano resonances and the LSPR splitting (Figure 2). In the following, we will focus on the LSPR nature of noble metal NPs and LSRP corresponding applications.

The various applications of plasmonic NPs are associated with two important physical effects. One is that the optical extinction of the metallic NPs has a maximum at visible near-infrared (NIR) wavelengths, which is much larger than their geometrical size. For example, Au NPs that are larger than 2 nm have approximatively 100% light-to-heat conversion efficiency, which is caused by their large extinction cross-section [22]. The heated NPs have many potential applications, such as the photothermal therapy in treating cancers [4]. The other is the localized enhancement of the electromagnetic field near the particles. In case the metallic spherical NPs have a size comparable to the metal skin depth (~100 nm), the electron can be driven by the light wave with a resonant frequency. As shown in Figure 1a, the electron gas oscillates similar to a dipole in the parallel direction to the electric field. Since the resulting plasmonic oscillation is distributed over the whole particle volume, the NPs act similar to a nanoantenna [23]. 

The optical properties of LSPR in an NP system, such as quality factor, resonance bandwidth, local field intensity, and so on, are influenced by many external and internal factors. For example, in a metallic NP dispersion, the measured optical extinction spectrum is the sum of absorbed and scattered photons. We can mathematically treat the extinction spectrum of NPs as the sum of the absorption and scattering terms:

(3) $$\sigma_{Ext}=\sigma_{Abs}+\sigma_{Sca}$$

The absorption and scattering of NPs are strongly dependent on the particle size. When the metallic NPs are much smaller than the wavelength of light (Figure 1c), the absorption becomes much stronger than the scattering. When the diameter of the NP is comparable to the incident wavelength, multipolar plasmon oscillation occurs inside the NP. As shown in Figure 1d, both absorption and scattering become comparable in this case. Thus, the size of NPs leads to the change of the optical constants of NPs [8]. 

The LSPR intensity can be quantified by the quality factor. Following Equation (2), the quality factor of plasmonic NPs, *Q*_NP_, can be written as [1]:

(4) $$Q_{\mathrm{NP}}\left(\omega \right)=-\frac{Re[\varepsilon (\omega )]}{Im[\varepsilon (\omega )]},$$

For a given resonance frequency *ω*, the resonance quality factor of a gold nanosphere depends only on the real and imaginary parts of its dielectric constant. A higher *Q*_NP_ factor corresponds to a spectrally narrowband and stronger local field enhancement. When the mean free path of conduction electrons is comparable to the particle size, the scattering by the free electron at the particle surface is immeasurable. Specifically, when the particle size is smaller than 30 nm, *Q*_NP_ decreased with the NP size. On the other hand, the permittivity of LSPR system can be controlled by the dielectric environment around the NPs. Following Equation (4), the change of *ε*(*ω*) leads to variation of the resonance frequency and *Q*_NP_ [8]. The polarizability dependence of the Au NPs on the frequency is shown in Figure 1b. The resonance frequency is redshifted with the increased *Re*[*ε*(*ω*)], and the NP extinction cross-section is increased at the same time. For the absorbing dielectric environment, the geometry of the absorbing materials and the coupling distance play a particularly prominent role. The imaginary part of the permittivity will weaken or even eliminate the LSPR resonance [23]. 

When the gold NPs are close to each other, the plasmon resonances are coupled to produce a variety of optical modes (Figure 2a). For specific NP assembly geometries, which are typically associated with well-defined NP disposition and homogeneous nanometric gaps between particles, the mutual interaction between plasmon oscillations can originate constructive and destructive interference phenomena yielding to asymmetric LSPR band shapes, and sharp dips or spikes in the optical extinction spectrum, which are known as Fano resonances [24]. It has been reported that the strong coupling can be achieved by the Au NP on the mirror system, which has a ~1 nm gap between the NP and the metallic mirror [25]. Figure 2b–d show the strong coupling of a single molecule in plasmonic nanocavities at room temperature. Since the plasmonic resonance mode volume is less than 40 nm^3^, remarkable energy-level splitting is achieved in ordinary ambient conditions where there is no low temperature and no high-Q optical cavity. In addition, non-ambient conditions such as low temperature, high pressure, etc., can also have significant impact on the characteristics of the LSPR [26,27].

### 2.2. Applications of SPR in Au NPs

When the free electrons in the NPs resonate with the incident electromagnetic field, a strongly localized and enhanced electric field is created in the subwavelength regime. As a result, the NP acts as a nanolens to focus the electromagnetic field. This principle provides a method to enhance several types of optical phenomena in the nearby molecules or nanomaterials, such as Raman scattering [11,28], photoluminescence, extinction, and nonlinear effects [29]. The enhancement factor near the metallic NP surface can be expressed as *EF = E*_loc_/*E*_0_, where the *E*_loc_ and *E*_0_ are the local and incident electric field, respectively. The *EF* can reach 10^2^–10^3^ in most LSPR cases by using the metallic NPs [11]. For similarly sized nanospheres and nanotetrahedrons, the *EF* of nanospheres is smaller than the nanotetrahedrons because the nanotetrahedrons have sharper edges or tips, which allow free electrons to localize at the tip to produce a much more intense field [2]. In case of an NP dimer or trimer, the largest *EF* is possible as the junctions occur between NPs [4].

#### 2.2.1. Surface Enhancement Raman Scattering

**Figure 3:**
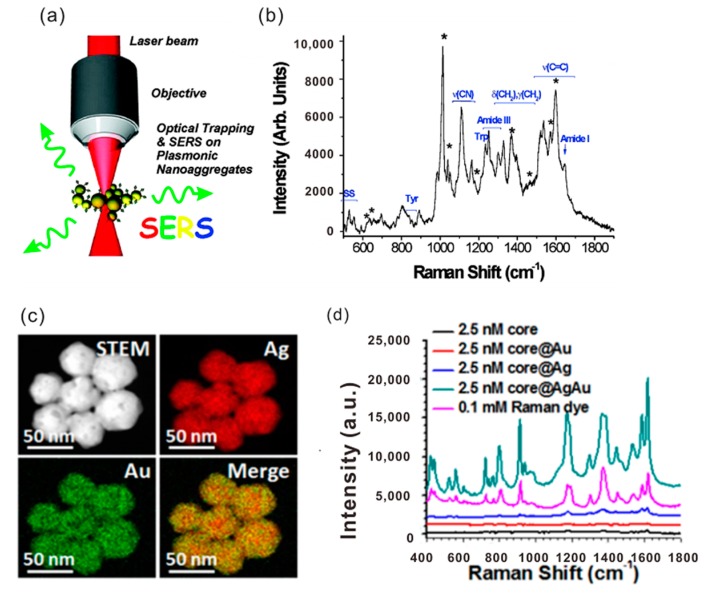
(**a**) Schematic of the Raman tweezing. Adapted from [30], with permission from © 2011 American Chemical Society. (**b**) Raman signals from the pyridine and bovine serum albumin adsorbed on the trapped Au NPs. (**c**) Elemental mapping of core@AgAu nanoparticles using high-resolution transmission electron microscopy. (**d**) Raman intensity of core, core@Au, core@Ag, core@AgAu NPs, and Raman dyes. Core represents 16-nm Au NPs coated with methoxy poly(ethylene glycol) thiol (mPEG-SH), Raman dyes, and Pluronic F127. Adapted from [11], with permission from © 2016 American Chemical Society.

The far-field radiation by LSP is well-employed in the surface-enhanced Raman scattering (SERS). Figure 3a displays a schematic of SERS by Au NPs. If the LSPR occurs beside the Raman scattering molecules, the low efficiency of Raman scattering can be strongly improved, which represents the structure effect of the plasmonics (Figure 3b). The total SERS enhancement factor *E*_SERS_ depends on two terms as *E*_SERS_ = *E*_em_*E*_chem_, where *E*_em_ and *E*_chem_ account for the increase of the surface Raman signal due to the electromagnetic effect and the chemical effect, respectively [11,30]. Commonly, the *E*_em_ term can reach values as high as ~10^10^ inside a hot spot with the smallest mode resonance volume. However, the average *E*_em_ over the whole Au NP surface leads to the reduced enhancement. Experimentally measured SERS results show that the averaging *E*_em_ can reach 10^7^ by an Au NP substrate. *E*_chem_ is normally less than 10^2^. The chemical enhancement is formatted by the charge-transfer state between the NP surface and the molecule, which can be tuned by the excitation laser. 

SERS gives rise to the applications in many fields such as physical and bioanalytical chemistry, nanomedicine, and biomarkers (Figure 3c). It is worth noting that advances in substrate design, miniaturization, and device sensitivity are driving SERS toward new applications. In short, SERS is a well-established vibrational spectroscopy technology that can provide both fingerprint recognition capabilities and extremely high detection sensitivity down to the single-molecule level [31]. 

#### 2.2.2. Fluorescence Modulation

The nearby presence of Au NPs can strongly influence the fluorescent emission of a molecule or a fluorescent nanostructure. The fluorescence of the emitter is proportional to the fluorophore absorption and its quantum yield. The quantum yield of an emitter is its intrinsic property, and the fluorescence enhancement factor can be expressed as *EF*_fluo_ = *σ*_NP_/*σ*_Fluo_, where *σ*_Fluo_ is the fluorophore absorption cross-section [31]. Therefore, *EF*_fluo_ depends on the enhancement of the absorption cross-section and the enhancement/quenching undergone by the quantum yield. *EF*_fluo_ can reach values up to 1000, but it can also be less than one [31]. 

Fluorescence modulation has been used in the detection of metal ions, small molecules, proteins, and mammal cells. The displacement of the label by interaction with the analyte originated a measurable decrease or increase of the fluorescence signal. For example, fluorescence enhancement was achieved by coating a silica layer of well-defined thickness of the Au NP surface [32]. The combination of light-sensitive organic molecules and Au NPs are often used as dynamic plasmonic devices to control fluorescence enhancement or loss [33].

#### 2.2.3. Optical Trapping and Manipulation

**Figure 4:**
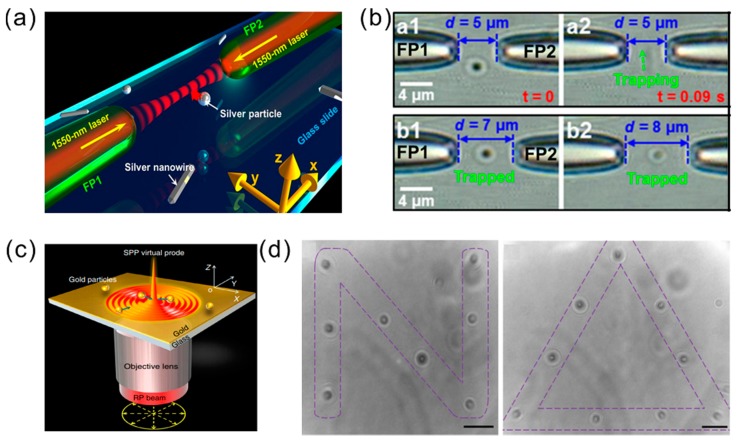
(**a**) Schematic for three-dimensional (3D) optical trapping silver nanostructures. (**b**) Optical trapping images obtained using a tip-to-tip fiber couple. Adapted from [35], with permission from © 2016 Springer Nature. (**c**) Schematic of trapping metallic particles by an surface plasmon–polariton (SPP) virtual probe. (**d**) Patterns constructed by Au NPs in the focused plasmonic tweezers. Adapted from [36], with permission from © 2013 Springer Nature.

Optical trapping is one of the most important modern-day techniques for nanoscience. It has many medical and micro/nanoproduction applications. The optical tweezer (OT) is a powerful tool based on a focused laser spot that can trap and manipulate micro-sized particles. OT based on plasmonics can give the molecule manipulation on an optical diffraction-limited scale. The suitable plasmon resonant frequency and polarization direction for the plasmonic OT is required to achieve the trapping and manipulation of Au NPs [34,35]. As shown in Figure 4a,b, the metallic NPs or NRs can also be pushed along the laser beam propagation axis, as the frequency is close to the LSPR region. Thus, plasmonic tweezers based on LSP provide the tools of three-dimensional (3D) optical trapping for nanoscale materials under the optical diffraction limited [34]. In coupled pairs of Au NPs on the substrate, the generated strong local field of LSP traps the nanoscale molecules between two NPs. NP pair arrays can not only improve the capture efficiency, they can also significantly reduce the laser energy requirements. Recently, the interference of surface plasmons from the gold film demonstrated a new type of small-scale OT that can arrange metal NPs neatly (Figure 4c,d) [36].

#### 2.2.4. Sensing and Imaging with Plasmon Resonance of NPs

**Figure 5:**
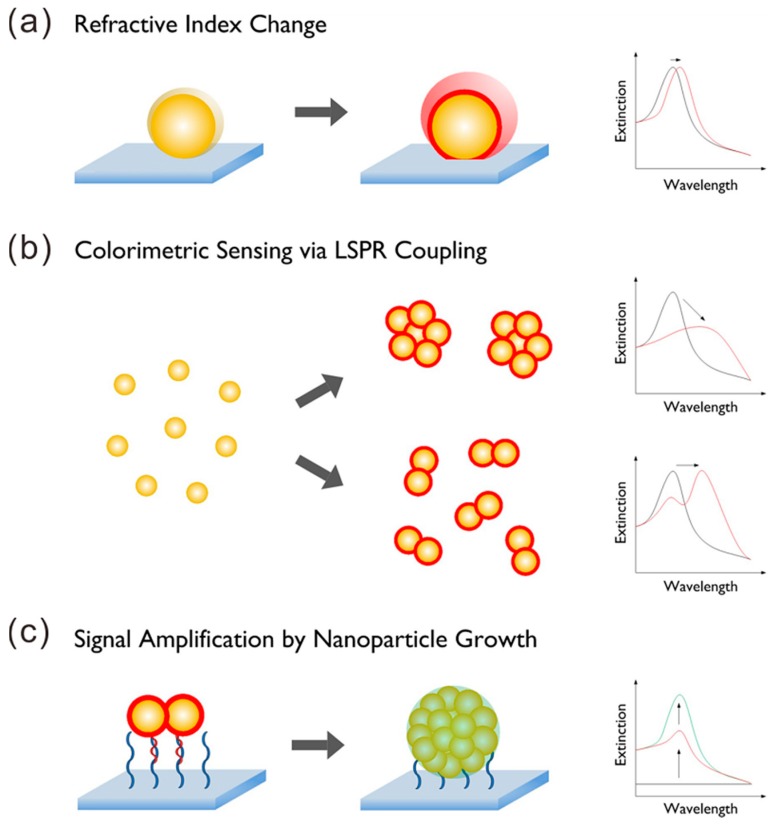
Illustration of different strategies to improve the sensitivity of plasmonic nanosensors. There are several types of methods that are available to influence the local dielectric properties surrounding plasmonic NPs that are motivated by factors such as (**a**) NP characteristics, (**b**) plasmonic coupling, and (**c**) amplification schemes. Three spectral graphs on the right of the figure give a sign for the change of the extinction spectra with the NP state. Adapted from [39], with permission from © 2015 Elsevier.

Due to the enhanced extinction cross-section and the tunability of plasmon resonance by the surrounding chemical environment of Au NPs, Au NPs become an excellent signal converter and are widely used for sensor development. Au NPs have many benefits, such as high chemical and biological stability, high sensitivity by the high surface-to-volume ratio, easy probe, and so on [37]. There are two main classes of optical sensors based on NPs. One is colorimetric sensing with assemblies of NPs, and another one is sensing connected to the LSPR spectral shift. Figure 5 shows the working principles of three sensors based on the LSPR effect of NPs. As shown in Figure 5a, efficient sensing is based on the local refractive index change of the medium surrounding the NP surface. individual gold particles covered by different analytes indices lead to a significant colorimetric phenomenon [38]. As shown in Figure 5b, the binding of the analyte on the NP surface can induce the aggregation of the NPs, resulting in interparticle surface plasmon coupling that translates into a visible change of color [39]. Another When the NP is fixed on a substrate such as a glass and a metal film instead of being in a solution, the performance of the sensor is greatly improved. In this case, the detection relies in the UV-VIS spectroscopy. Thus, the signal intensity can be easily distinguished. For example, the sensor can detect changes of the ion concentration around gold NPs in real time [40].

## 3. Plasmon-Enhanced Upconversion Luminescence 

Lanthanide-doped upconversion nanoparticles (UCNPs), which are capable of converting low-energy excitation light [e.g., the near-infrared or infrared (NIR or IR)] into high-energy emission (e.g., ultraviolet, visible, or NIR), have shown a wide range of potential applications in lighting and displays, bioimaging, therapeutics, and photovoltaic devices due to their excellent photostability, large anti-Stokes shift, narrowband emission, and long excited-state lifetime [41,42,43]. However, the low upconversion luminescence (UCL) efficiency of UCNPs is one of the major bottlenecks hindering their further potential applications. Recent experimental and theoretical studies have led to the development of several approaches to enhance UCL, including the core–shell structures, energy transfer modulation, photonic crystal engineering, and plasmonic enhancement effect [44,45,46,47,48,49]. Among these methods, the electric field enhancement via plasmonics offers a promising way of enhancing the UCL efficiency, and has aroused extensive attention.

The plasmon enhancement effects on UCL involve various processes including the absorption, emission, and energy transfer processes, as described below [45,48,50]. Briefly, they are the (1) absorption enhancement of the sensitizer through the localized optical field, (2) modulation of the decay rate, emission intensity, spectral profile, and quantum yield via the Purcell effect, and (3) enhanced energy transfer process from the donors to acceptors. Up until now, the UCL EF in the literatures varies in the range of several to thousands of folds.

### 3.1. Plasmon-Enhanced Absorption

The LSPR effect enables the enhancement of the local electric field in the vicinity of the metallic nanostructures, and then results in the increased absorption of the sensitizer ions, consequently enhancing the emission intensity of UCNPs without much effect on the quantum yield and lifetime decay of the UCNPs. In previous reports [47,50,51,52,53,54,55,56,57,58], various gold or silver nanostructures, such as NRs, NPs, nanowires, and the periodically patterned structures (including the NP arrays) have been explored for the plasmonic enhancement of UCNPs. In recent years, considerable efforts have been made on tuning the plasmon resonance wavelength to coincide with the excitation wavelength region. In the majority of the cases, it could significantly enhance the NIR absorption cross-sections of lanthanide dopants such as Yb^3+^ or Nd^3+^ by matching the LSPR of the metallic nanostructures with the NIR excitation at 808 nm and/or 980 nm. 

**Figure 6:**
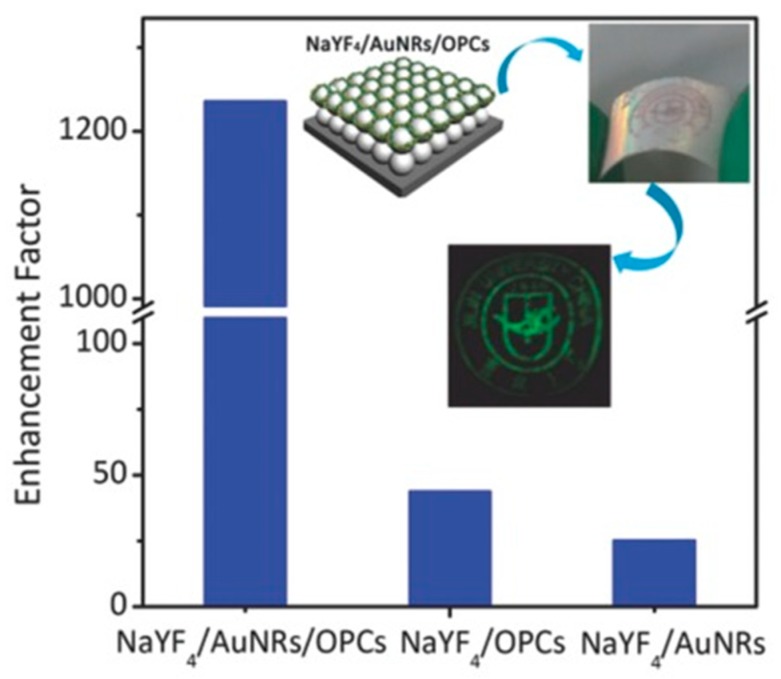
Enhancement factor of NaYF_4_/Au nanorods (NRs)/opal photonic crystals (OPCs), NaYF_4_/OPCs, and NaYF_4_/Au NR hybrids. The insets: schematic illustration of NaYF_4_/AuNRs/OPCs hybrids, optical image of flexible OPCs on a polyethylene terephthalate (PET) film, and a digital image of a flat flexible upconversion nanoparticles (UCNPs) film taken in a dark room under the excitation of a 980-nm laser. Adapted from [47], with permission from © 2016 John Wiley and Sons.

Typically, the anisotropic gold NR could incite two modes of plasmon resonance that are based on the polarization of the incident light. The longitudinal resonance mode is induced by the electric component along the long axis of a NR, and the transverse resonance mode is induced by the electric component across the long axis of a NR. Thus, the Au NRs are commonly used to achieve the tunable longitudinal resonance mode from visible to NIR via adjusting the aspect ratio (length/width) to gain a spectral overlap with the excitation band [59]. The Song group [47] combined the LSPR absorption of Au NRs and photonic crystal effects of three-dimensional (3D) polymethylmethacrylate (PMMA) opal photonic crystals (OPCs), and fabricated the NaYF_4_/Au NRs/OPCs hybrids’ structure. The results in Figure 6 shows the enhancement factor (EF) up to 1200-fold enhancement in the overall UCL intensity of Er^3+^, with 3000 and 600 times increase in the red and green emissions, respectively. Both theoretical and experimental analysis confirmed that the LSPR interacts strongly with the excitation electric field, leading to the large enhancement of the localized excitation field strength. In 2015, Song group also [51] introduced a vertically aligned gold NRs array monolayer to magnify the UCL intensity of NaYF_4_:Yb^3+^ and Er^3+^-doped UCNPs through a strong coupling of the SPR of gold NRs with the excitation field of the UCNPs. It led to an overall UCL enhancement up to more than 35-fold. Based on the above results, periodic structures exhibit well-defined coupling conditions for the irradiating light to achieve the absorption enhancement. Moreover, silver NPs have also been explored for the enhancement of the localized excitation field strength. Kwon et al. [52] applied the disordered array of silver NPs in the metal–insulator–metal (MIM) structure to harvest very high UCL enhancement, which was mainly due to the boosted absorption efficiency, and achieved an unprecedented EF of up to 1.35 × 10^3^. 

#### 3.1.1. Plasmon-Enhanced Emission 

Alternatively, plasmon-enhanced upconversion could also be achieved via overlapping the LSPR with the UCL emission wavelengths in the plasmonic UCNPs systems. With the introduction of plasmonic structures, the radiation transition rate of the luminescent center in UCNPs increases, leading to an enhancement of quantum yield and emission intensity, while the fluorescent lifetime decreases [46,53,54,55,56]. In previous works, various plasmonic UCNPs geometric configurations have been proposed to study the plasmon-enhanced emission. The core–shell structure is one of the most effective approaches to gain better control over the local environment of the lanthanide ions. In 2010, Zhang et al. [53] designed the gold NP shell onto the UCNP surface to acquire an increase of emission intensity. Since the upconversion emission overlapping with the plasmonic resonance band observed with gold NPs, they suggested that plasmon-enhanced emission played an important role in the spectrum-dependent enhancement of upconversion emission, which can accelerate the radiative decay rate and increase the emission efficiency. Meanwhile, the gold NP shell directly attached to the UCNP surface may also suppress the emission due to the quenching.

Accordingly, adding an isolation layer is an effective approach to control the gap between the UCNPs and the plasmonic nanostructures, and then alleviate quenching and tune the upconversion emission. As a reliable spacer, SiO_2_, undoped NaYF_4_, and Al_2_O_3_ shells have been widely used to achieve an emission enhancement in the core–shell plasmonic UCNPs nanostructures. For example, UCL enhancement of 6.7-fold, 14.4-fold, and 30-fold has been obtained for Au NR@SiO_2_@CaF_2_:Yb^3+^, Er^3+^, NaYF_4_:Yb, Er@SiO_2_@Ag, and Ag@SiO_2_@Lu_2_O_3_:Gd/Yb/Er [54,55,56] metallic nanostructures, respectively, which could be mainly attributed to the increase in the radiative decay rate and emission efficiency caused by the plasmon-enhanced emission. 

**Figure 7:**
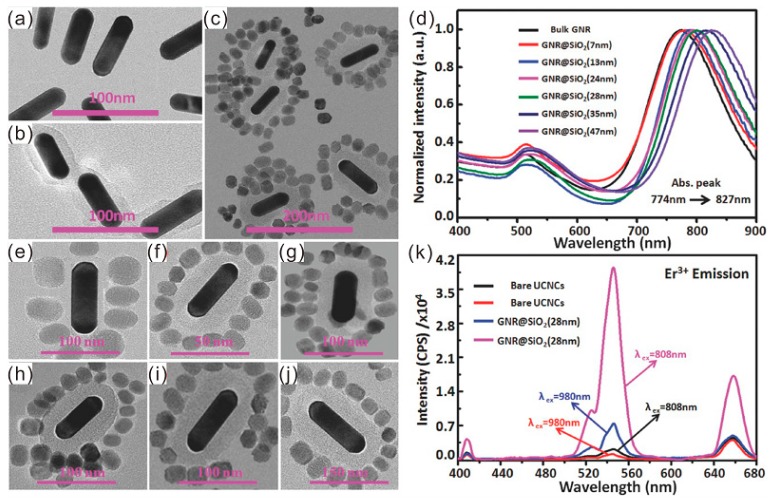
Low-magnification TEM images of as-prepared bare (**a**), SiO_2_-coated Au NRs (**b**), and hybrid AuNR@SiO_2_@NaGdF_4_:Yb, Nd@NaGdF_4_:Yb, Er@NaGdF_4_ nanostructures with 28-nm SiO_2_ thickness (**c**). (**d**) Measured extinction spectra of AuNR@SiO_2_ core–shell nanostructures with SiO_2_ shell thickness varied from 0 to 47 nm; (**e**–**j**) TEM images of single hybrid AuNR@SiO_2_@NaGdF_4_:Yb, Nd@NaGdF_4_:Yb, Er@NaGdF_4_ nanostructures with SiO_2_ thickness varied from 7 nm to 47 nm; (**k**) Measured luminescence spectra for bare UCNPs and hybrid nanostructures of 24-nm SiO_2_ shell under 808 nm (black and pink) and 980 nm (red and blue) continuous wave (CW) laser excitation. Note that the TEM image in (i) is part of the low-magnification TEM image in (**c**). Adapted from [58], with permission from © 2017 John Wiley and Sons.

It is worth noting that numerous works demonstrate both plasmon-enhanced absorption and emission work collectively together for the UCL enhancement, especially in the anisotropic gold NR systems. Since the longitudinal resonance mode has a better spectral overlap with the absorption wavelength of UCNPs in the NIR region, while the transverse resonance mode could match well with the visible emission band of UCNPs, 10-fold and 20-fold UCL enhancements in the Au NR@mSiO_2_@Y_2_O_3_:Er [56] and Au NR@SiO_2_@UCNPs [57] were shown, respectively. Lei et al. have designed and fabricated a hybrid plasmonic upconversion nanostructure, which consisted of Au NRs@SiO_2_ surrounded by the UCNP structure (NaGdF_4_:Yb^3+^, Nd^3+^@NaGdF_4_:Yb^3+^, Er^3+^@NaGdF_4_ core–shell–shell structure) [58]. The thickness of the SiO_2_ spacer was controlled from 7 nm to 47 nm, as shown in Figure 7, resulting in a gradual redshift in the longitudinal resonance mode from 774 nm to 827 nm, while the transverse resonance mode remains relatively unchanged at 530 nm, consequently achieving an emission dual enhancement and flexible color tuning in the UCL through adjusting the distance between the gold NR and the UCNP. A 2.83-fold, 18.85-fold, and 4.87-fold increase in the enhancement factors for the blue, green, and red emission of the hybrid plasmonic upconversion nanostructure can be observed upon excitation at 808 nm, when the thickness of the SiO_2_ spacer was optimized to 28 nm. With the experimental and simulation results, they also attributed the UCL enhancement to the longitudinal and transverse resonance excitation and emission enhancement of the nanostrucutres. 

#### 3.1.2. Plasmon-Enhanced Energy Transfer

**Figure 8:**
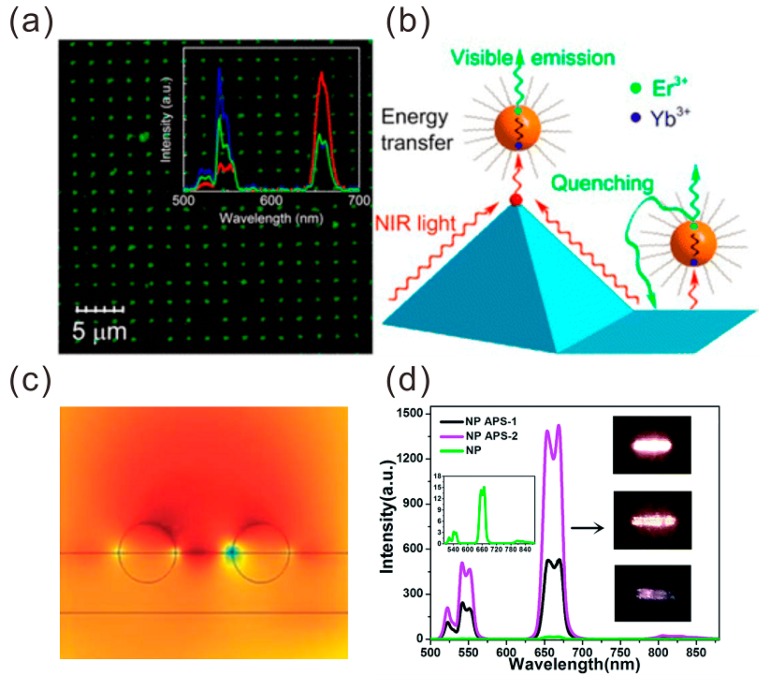
(**a**) Confocal images for the green emission on 29-nm NaYF_4_:Yb^3+^/Er^3+^ particles. (**b**) Schematic of the energy transfer, upconversion, and quenching processes on the top and bottom of the gold pyramid substrate. The ultrasmooth gold pyramid provides a good platform to study all of these photophysical processes in the doped-lanthanide NPs. Adapted from [59], with permission from © 2014 American Chemical Society. (**c**) Simulated electric field distributions. (**d**) Comparison of emission spectra of NaYF_4_:Yb^3+^ and Er^3+^ NPs deposited on the quartz substrate (green line), Au papilla surface type 1 (APS-1) (black line), and Au papilla surface type 2 (APS-2) (pink line). Insets are the optical images of the NaYF_4_:Yb^3+^ and Er^3+^ NPs spin-coated on the quartz substrate (down), APS-1 (middle), and APS-2 Au films (up). Adapted from [60], with permission from © 2016 RSC Publishing.

The vast majority of literatures have reported the plasmon-enhanced upconversion via the enhancement of energy transfer. Generally, the energy transfer mainly originated between the interaction between the dipole in the donor from the UCNPs and the free electrons in the acceptor of the metallic structures. In 2013, Sun et al. [59] fabricated the gold pyramid pattern to increase the rate of resonant energy transfer from Yb^3+^ to Er^3+^ ions by six times, as shown in Figure 8a,b, and proposed that the energy transfer may be ascribed to the strong Coulombic effect, while, as a contrast, they also found the strong metal mediated quenching (14-times) of green fluorescence on flat metal surfaces. In addition, Wang et al. [60] reported the enhancement of NaYF_4_:Yb^3+^ and Er^3+^ NP UCL by using two kinds of Au films for tunable and broad surface plasmonic absorptions. The results showed that the UCL enhancement is highly dependent on the topography of Au films. About 77-fold and 40-fold enhancements were obtained for the green and red UC emissions of NaYF_4_:Yb^3+^ and Er^3+^ NPs on the irregular and random Au particles. About 121-fold and 78-fold enhancements were obtained for the green and red UC emissions of NaYF_4_:Yb^3+^ and Er^3+^ NPs on continuous Au films with papilla Au NPs, respectively. As shown in Figure 8c,d, in contrast to that of NaYF_4_:Yb^3+^, Er^3+^ NPs deposited on quartz substrate, and the corresponding UC efficiency of NaYF_4_:Yb^3+^ and Er^3+^ NPs on irregular and random Au particles and continuous Au films with papilla Au NPs increased by 50% and 100%, respectively.

In some plasmonic UCNP nanostructure systems, one of the above three types of enhancement will play the dominant role in the luminescent enhancement, while as suggested previously, the plasmon-enhanced absorption, energy transfer process, and emission will also cooperate together and contribute to the luminescent enhancement in some specific plasmonic UCNP nanostructure systems. Although the above three categories have been made to explain the plasmon-enhanced upconversion luminescence in energy transfer upconversion, a precise model and deep understanding of the mechanisms of LSPR enhancement UCL require further exploration.

### 3.2. Applications of Plasmonic UCNPs Systems

Recent years have witnessed a significant advance in the coupling effect of plasmonic UCNPs systems, which offers a new way to enhance the upconversion emission and luminescence efficiency of UCNPs, and combine with some certain properties of metallic nanostructures, and consequently paves the road to wide applications including lighting and displays, bioimaging, biodetection, photothermal therapy (PTT), photodynamics therapy (PDT), and so on [46,49,56,58]. Especially, since Au NPs and Au NRs have been demonstrated as good heat producers [46,61], plasmonic UCNP nanostructures with dual functions or multifunctions have attracted much attention for the potential applications in the biological field. Chen et al. [62] designed and fabricated UCNP@SiO_2_-Au NPs and UCNP@SiO_2_-Au NRs nanostructures to combine the dual function plasmon-enhanced UCL and photothermal properties of metallic nanostructures for the potential application of bioimaging and PTT. With the modified surface with folic acids, the hybrid structures were tested for the treatment of OECM-1 oral cancer cells via PTT and cell imaging. In 2017, Huang et al. [63] also observed the upconversion emission enhancement and photothermal property in Au NR@SiO_2_@UCNPs nanocomposites with integrated functionalities, including bioimaging and PTT. Meanwhile, the photosensitizer, zinc phthalocyanine (ZnPc), was introduced into the nanocomposite and excited by the visible light generated from the UCNPs, thereby triggering the release of reactive oxygen species for application in the PDT of cancers. While numerous experimental techniques and structures have been studied for the plasmon enhancement upconversion, plenty of room still exists for a wide range of potential applications with the novel design of plasmon upconversion nanostructures.

## 4. Chirality Plasmonic Metasurface

As a branch of plasmonics, chiral plasmonics has attracted tremendous attention for its intriguing properties and vast applications. In nature, chirality is ubiquitous, and can be observed at various scales ranging from molecules to amino acids, proteins, and the shape of galaxy. In optics, chiral objects can show different responses to right or left-handed circularly polarized light (CPL). These phenomena are typically called chiroptical effects. One of most well-known chiroptical effects is circular dichroism (CD), which has wide applications in chemistry, biology, and pharmacy. In recent years, optical chiral metamaterials have attracted remarkable attention as exciting opportunities for fundamental research and practical applications. Along this line, one can design artificial plasmonic chirality by the advanced nanofabrication techniques. Compared with natural chiral materials, artificial chiral metamaterials exhibit many intriguing properties, such as strong CD, a chiral negative refractive index, asymmetric transmission, and superchiral fields [64,65,66,67,68,69,70,71,72,73,74,75,76]. The local chiral field can be enhanced by the LSPR to drive a wide range of physical and chemical processes [77].

In general, the fabrication of chiral plasmonic metasurfaces can be classified by two approaches: top–down and bottom–up methods. The top–down approach uses various nanofabrication techniques to cut, etch, and engrave the materials into the desired chiral metamaterials. The bottom–up approach usually make the chiral metamaterials by assembling, stacking, and arranging small components.

### 4.1. Chiral Metamaterials Based on Top–Down Fabrication Methods

**Figure 9:**
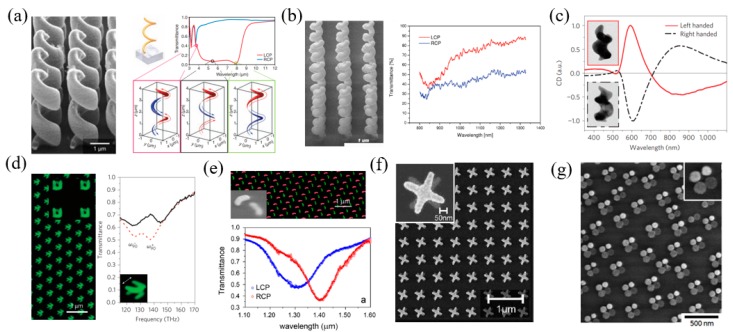
Chiral metamaterials fabricated by the top–down method. (**a**) Plasmonic helices fabricated by two-photon direct laser writing. The simulated transmittance spectra and plasmonic modes are depicted in the left panel. The incident LCP light is totally blocked, while RCP light is transmitted nearly without loss. Adapted from [65], with permission from © 2009 American Association for the Advancement of Science. (**b**) Helicoidal three-dimensional nanostructures fabricated by focused ion beam-induced deposition method; the spectrum shows strong chirality. Adapted from [72], with permission from © 2015, American Chemical Society. (**c**) Two symmetric pairs of gold spirals fabricated by the glancing angle deposition method, which exhibit a strong circular dichroism (CD) response in the visible wavelength range. Adapted from [73], with permission from © 2013, Springer Nature. (**d**,**e**) Field emission electron microscopy and transmission spectra of a double-layered twisted gold split ring (**d**) and twisted arc (**e**) metamaterial. (**d**) Adapted from [76], with permission from © 2009, Springer Nature; (**e**) Adapted from [78], with permission from © 2014, American Chemical Society; (**f**) Twisted stacked crosses. Adapted from [79], with permission from © 2009 The Optical Society of American. (**g**) Four particles of different heights form a chiral structure. Adapted from [81], with permission from © 2014, American Chemical Society.

The top–down method includes direct laser writing [65,66,67,68,69], focused ion beam lithography [70,71,72], glancing-angle deposition [73,74], and electron beam lithography [76,77,78,79,80]. One earlier typical chiral nanostructure is a helix or spiral with intrinsic twist. For example, Gansel et al. proposed a plasmonic metamaterial composed of gold helices [65], which were fabricated by two-photon laser writing and the subsequent electrochemical deposition of gold. Such helices exhibit plasmonic resonant modes that extend through the whole structure, as shown in Figure 9a. The resonant modes show distinct handedness, and mainly interact with the same handed CPL. When CPL with the same handedness illuminates on the metamaterial, the plasmonic modes are induced, and light is transmitted. In contrast, CPL with the opposite handedness cannot induce these modes, and then it is blocked by the metamaterial. As expected, the authors demonstrated that the metamaterial shows a broadband transmittance spectrum for one-handed CPL in the spectral range from 3.5 um to 7.6 um. Therefore, the proposed metamaterial could work as a broadband circular polarizer to convert the incident linearized light to CPL. The metallic nanospiral structures can be also fabricated by the focused ion beam induced deposition method. Esposito et al. employed this method to fabricate platinum helicoidal three-dimensional nanostructures as broadband chiral metamaterials in the near infrared region, as shown in Figure 9b [72]. The handedness of the nanospirals was controlled by the sweeping direction of the ion beam. The surface charge effects on substrates with different electrical properties can influence the growth of the nanostructures and induce a dimensional gradient of the wire diameter of the nanospirals. The experimental results showed a transmittance difference of the right and left circularly polarized (RCP and LCP) light near 40%. The glancing angle deposition is another efficient method to fabricate 3D chiral nanostructures, which are demonstrated by many research groups. For example, Mark et al. fabricated gold nanohelices as 3D chiral nanostructures, leading a to strong chiroptical effect. The left side of Figure 9c shows the transmission electron microscopy (TEM) images of the fabricated helix. The measured CD spectra [73] on the right side of Figure 9c for both helices with opposite rotation (also called enantiomers) show a strong chiral response around 600 nm, and they are just symmetric.

The above-mentioned chiral structures are real spirals that have intrinsic handedness. Layered planar nanostructures for chiral plasmonic structures have been fabricated. The unit cells of the planar structures could be split ring resonators (SRRs) [76], gammadions [77], arcs [78], or any other structures. These structures could be fabricated layer-by-layer by using electron beam lithography. Figure 9d shows an example of a double-layered metasurface composed of twisted SRR [76]. When the free electrons are excited by the incident light at a specific resonant frequency, the equivalent current distributions of the U-shaped nanostructure include an inductances loop and a capacitance loop. For single-layered SRR nanostructures, they are achiral on their own. When two layers of such nanostructures are placed together, their electric and magnetic dipoles can couple to each other, leading to a hybrid mode and chirality. The coupling effects depend on the twist angle between the two layers. As a result, the transmittance spectrum as well as the chiroptical effects can be tuned by adjusting the twisted angle. In particular, when the twisted angle is 90°, the magnetic dipole–dipole coupling between the two layers plays a dominant role. There are two hybrid modes with strong chiroptical effects, which are the parallel and antiparallel modes, respectively, and can be seen in the transmittance spectrum in Figure 9d. Similarly, following the same idea, other double-layered nanostructures such as arcs (Figure 9e) [78] and crosses (Figure 9f) [79] also showed very strong chirality. Another interesting example is shown in Figure 9g. Although the four particles are in an achiral arrangement, they have different heights, which can be verified by their different brightness. This structure can support plasmonic coupling modes and generate a pronounced chirality [81].

### 4.2. Chiral Metamaterials Based on Bottom–Up Fabrication Methods

**Figure 10:**
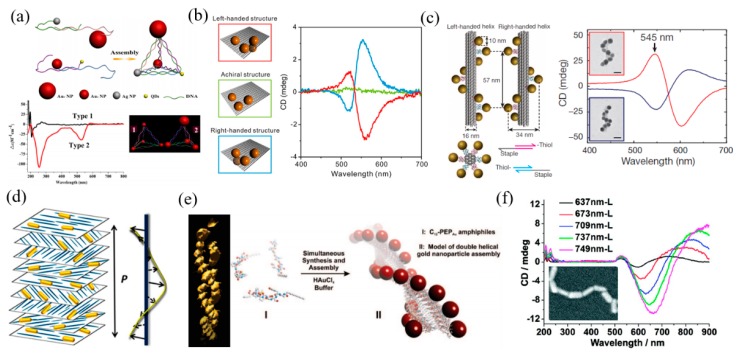
Nanostructures fabricated by bottom–up methods. (**a**) Schematic of chiral NP pyramids constructed by DNA scaffolds and their CD spectra. Adapted from [82], with permission from © 2012 American Chemical Society. (**b**) Four Au NPs assembled with an origami sheet with four docking sites. Three of the docking sites are along an L-shape on the one interface and one on the other. The NPs form left-handed, right-handed and achiral structure system and the corresponding CD spectra. Adapted from [85], with permission from © 2013 American Chemical Society. (**c**) Schematic of plasmonic nano helices with both left and right handedness based on of DNA self-assembly and the corresponding CD spectra. Adapted from [86], with permission from © 2012 Springer Nature. (**d**) Chiral gold NRs system synthesized by a layered, twisted cellulose nanocrystals template. Adapted from [87], with permission from © 2014 American Chemical Society. (**e**) Schematic of the formation of Au NP double helices, which are synthesized and assembled directly in a reaction containing HEPES buffer solutions of chloroauric acid and C_12_-PEP_Au_. Adapted from [89], with permission from © 2008 American Chemical Society. (**f**) SEM image of the one-dimensional (1D) self-assembled gold NRs (GNRs) and their CD spectra. Adapted from [90], with permission from © 2012 American Chemical Society.

Top–down fabrication methods have disadvantages, such as time consumption and high cost. The other route that is based on self-assembly technology, the so-called bottom–up method, provides the large-scale fabrication with a lower cost. Various templates have been used for the bottom–up fabrication of chiral nanostructures such as DNA [82,83,84,85,86], cellulose nanocrystals [87], cholesteric liquid crystals [88], cysteine [89], peptides [90], and so on. DNA self-assembly utilizes DNA scaffolding or DNA origami to connect plasmonic NPs. Yan et al. proposed a family of self-assembled chiral pyramids made from different metal NPs and quantum dots [82]. The DNA strands act as a scaffold for connecting the NPs with different sizes and materials and controlling their positions, as shown in Figure 10a. The optical properties and handedness of the pyramid four-particle systems can be easily tuned by changing the arm lengths and symmetry of the pyramid structures. Apart from the DNA scaffold, the DNA origami sheet can be also used for constructing the 3D chiral structures in such four-particle systems. Shen et al. utilized a bifacial rigid addressable DNA origami template to connect four gold NPs to form a chiral tetramer [85]. The left side of Figure 10b shows the experimental structures. The template has three docking sites on the top surface, and one sits on the bottom surface. Each site connects an identical gold NP, and the four particles form a 3D structure. As seen in the right side of Figure 10b, the 3D assemblies exhibit a strong chiral effect with pronounced CD spectra due to the strong plasmonic resonant coupling between the gold NPs and the chiral structural symmetry. The DNA origami templates are also efficient for the synthesis of 3D chiral nanostructures. Kuzyk et al. demonstrated a gold NR helix structure constructed from a folding DNA origami template [86], as shown in Figure 10c. The gold NRs are functionalized with complementary DNA strands, and are able to bond with the rectangular DNA template at predesigned positions. Then, the DNA template will roll up by using the folding strands, and the NR assemblies become 3D helical structures. The experimental results showed that the assemblies exhibit strong chiroptical effects around 545 nm. The spectra of left-handed and right-handed assemblies are complementary to each other. Apart from DNA templates, other templates have also been widely studied. The left side of Figure 10d schematically shows the 3D chiral gold NRs system that is synthesized by using cellulose nanocrystals (CNCs) [87]. The authors prepared chiral plasmonic films by incorporating gold NRs in a macroscopic cholesteric film formed by self-assembled CNCs. The CNCs have a cholesteric liquid crystalline phase in which adjacent CNC layers rotate anticlockwise with respect to each other and form a left-handed helical structure. Then, the CNC films work as templates, and are loaded with gold NRs. Composite NR–CNC films show pronounced plasmonic chirality, which are easily tunable by changing the conditions of film preparation. Using the same idea, templates based on cholesteric liquid crystals have been widely used for the synthesis of chiral nanostructures. Chen et al. prepared gold NP double helices based on peptides, as shown in Figure 10e [89]. The authors firstly prepared peptide conjugates, PEP_Au_, which have a high affinity for gold surfaces. Then, they coupled succinimide-activated dodecanoic acid to the N-terminus of PEP_Au_ to generate [C_11_H_23_CO]-PEP_Au_, or C_12_-PEP_Au_. Mixing them with a gold NP precursor solution and a HEPES buffer will form left-handed or right-handed double-helix structures, respectively, which show strong chirality. In another work, Zhu et al. synthesized the one-dimensional assembly of a cysteine (CYS) and gold NR (GNR) system [90]. As shown in Figure 10f, GNRs can form an end-to-end chain through the electrostatic attraction of CYS molecules, which are preferentially attached on the ends of different gold NRs. The wavelength of the CD responses can be manipulated by using light from UV and the visible-NIR region through changing the aspect ratios of gold NRs in 1D assembly.

## 5. Conclusions

The physics of plasmonics is rich and interesting. In recent years, noble metallic NPs have been the major research subject related to their LSPR properties. With in-depth studies of LSPR, theoretical models have been well established and applied to various LSPR cases. These theoretical advances have also led to design and applications in different fields, such as optical sensing, SERS and photothermally-induced optical trapping, enhanced catalysis and luminescence, etc. Theoretically, the enhancement of the localized field plays an important role among current applications. The local field enhancement, which relies on the size of the NPs, not only allows the optical detection beyond the diffraction limit, but also greatly enhances the far-field radiation of the weak signal. Therefore, nanoscale particles can be manipulated by the plasmonic tweezer, which is twice as small than the traditional OT. A weak Raman scattering signal can be amplified by 10^2^- or 10^3^-fold. Due to the selective frequency absorption, the NPs can efficiently catalyze the photochemical reaction. More than a 10^2^-fold enhancement has been obtained for the UCNP emissions due to the high energy-transfer efficiency of the NPs. Researchers have also developed plasmonic devices with unprecedented optical performance through precise nanofabrication. With the help of those advanced nanofabrication techniques, such as direct laser writing, focused ion beam lithography, glancing-angle deposition, and electron beam lithography, the chiral devices with the ultra-high resolution have demonstrated excellent optical response in high-frequency bands, which are of great importance in biological macromolecules and DNA-related studies.

The potential use of plasmonic devices is as ubiquitous as optics. These applications include new approaches to unresolved issues and improvements in sophisticated optical processes. Fundamental research will continuously lead to new potential applications in future. Current challenges for applications are the accurate and precise control of the size, shape, and functionalization of NPs. Especially, the large-scale, low-cost synthesis of uniformly sized and shaped NPs and excellent control of their dispersion are the keys to promoting a wide range of applications of plasmonic technology in the energy field. With the continuous theoretical advancement of plasmonics, as well as the parallel development of nanofabrication techniques, an exciting and blooming era of plasmonics begins with more interesting scientific challenges and real-life applications in the near future.
